# Supplementary material for: Comparative genomics provides new insights into the diversity, physiology, and sexuality of the only industrially exploited tremellomycete: Phaffia rhodozyma
Source: BMC Genomics. 2016 Nov 9;17:901. doi: 10.1186/s12864-016-3244-7 (PMC5103461; doi:10.1186/s12864-016-3244-7)
Supplement: Additional file 6: — List of orphan genes with links to PFAM (related to Additional file 1: Table S1). (ZIP 1428 kb) [file 12864_2016_3244_MOESM6_ESM.zip › PFAM_UNIQUE/G02662_P.html]

#                                                                            --- full sequence --- -------------- this domain -------------   hmm coord   ali coord   env coord
  
# target name        accession   tlen query name           accession   qlen   E-value  score  bias   #  of  c-Evalue  i-Evalue  score  bias  from    to  from    to  from    to  acc description of target
  
#------------------- ---------- ----- -------------------- ---------- ----- --------- ------ ----- --- --- --------- --------- ------ ----- ----- ----- ----- ----- ----- ----- ---- ---------------------
  
G02662\_P             -            601 DUF3210              PF11489.3    711       9.9    3.3   8.2   1   2      0.88   6.6e+02   -2.7   1.5    82    93   160   171    90   217 0.59 - Protein of unknown function (DUF3210)  
G02662\_P             -            601 DUF3210              PF11489.3    711       9.9    3.3   8.2   2   2   0.00027       0.2    9.0   0.7    74   151   415   487   374   502 0.76 - Protein of unknown function (DUF3210)  
G02662\_P             -            601 DUF4203              PF13886.1    210     0.017   13.3   0.0   1   1   1.7e-05     0.025   12.7   0.0   113   169    31    85    22    97 0.87 - Domain of unknown function (DUF4203)
